# Supplementary material for: Myelinating Co-Culture as a Model to Study Anti-NMDAR Neurotoxicity
Source: Int J Mol Sci. 2022 Dec 23;24(1):248. doi: 10.3390/ijms24010248 (PMC9820503; doi:10.3390/ijms24010248)
Supplement: Supplementary file 1 [file ijms-24-00248-s001.zip › ijms-2000469-supplementary.pdf]

Myelinating co-cultures as a model to study anti-NMDAR neurotoxicity.

Mercedeh Farhat Sabet[1], Sumanta Barman[1],Mathias Beller[2], Sven G. Meuth[1], Nico Melzer[1], Orhan Aktas[1], Norbert Goebels[1]* and Tim Prozorovski[1]*

[1]Department of Neurology, Medical Faculty, Heinrich-Heine-Universität Düsseldorf, Moorenstr. 5, Düsseldorf, 40225, Germany

[2]Institut für Mathematische Modellierung Biologischer Systeme, Heinrich-Heine-Universität Düsseldorf, 40225 Düsseldorf, Germany.

*These authors contributed equally to this work.

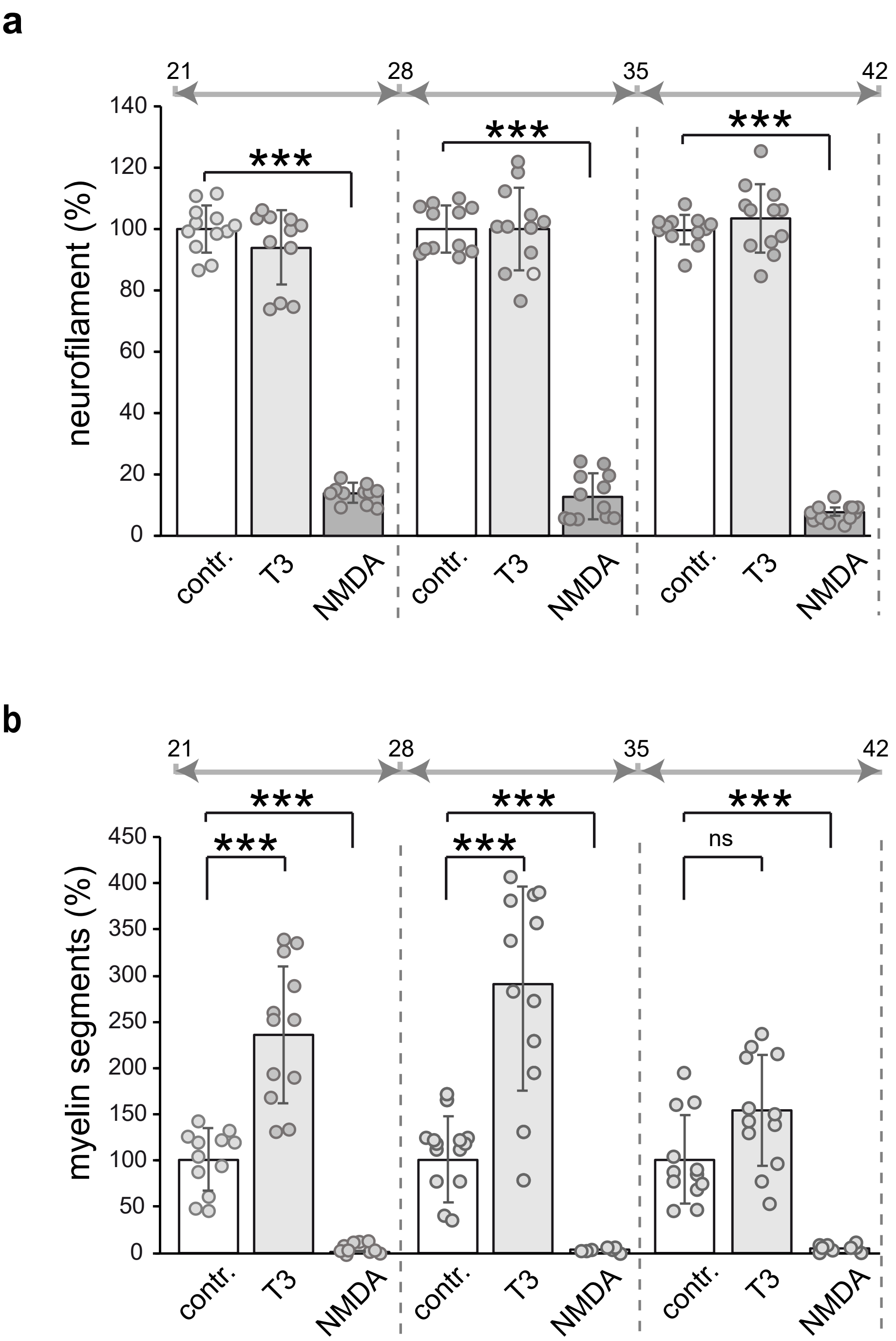

Figure S1. Responses to NMDA and T3 hormone treatment in myelinating spinal cord cultures. Cultures were treated for one week with NMDA (100 μM) or T3 (1 μM). (a) CellProfiler analysis of neurofilament immunoreactivity (immunostaining for NF200) revealed a drastic decrease of neurite density in the NMDA-treated group. (b) CellProfiler analysis of myelin segments (double immunostaining for NF200 and MBP) depicted increased myelination in T3-treated group and loss of myelination upon NMDA toxicity. One-way ANOVA with Tukey multiple comparison showed significant differences between T3 and NMDA vs control groups. Mean ± SEM, (n = 3; technical replicates = 4); ns, non-significant; *** $p < 0.001$.

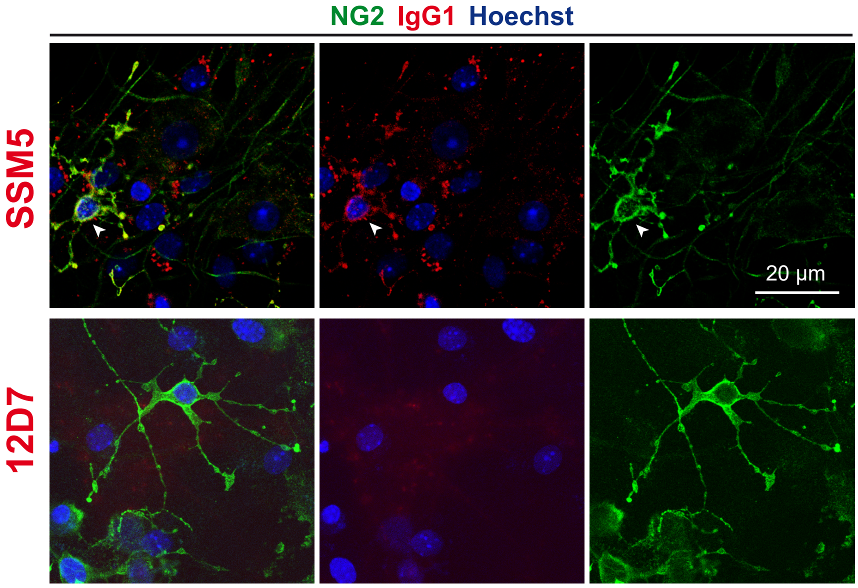

Figure S2. Immunofluorescence analysis of the reactivity of SSM5 in NG2-positive cells. Immunocytochemical localization of SSM5 (red) on cells stained for NG2 (green). Nuclei were counterstained with Hoechst 33258 (blue). Scale bar: 20 µm.

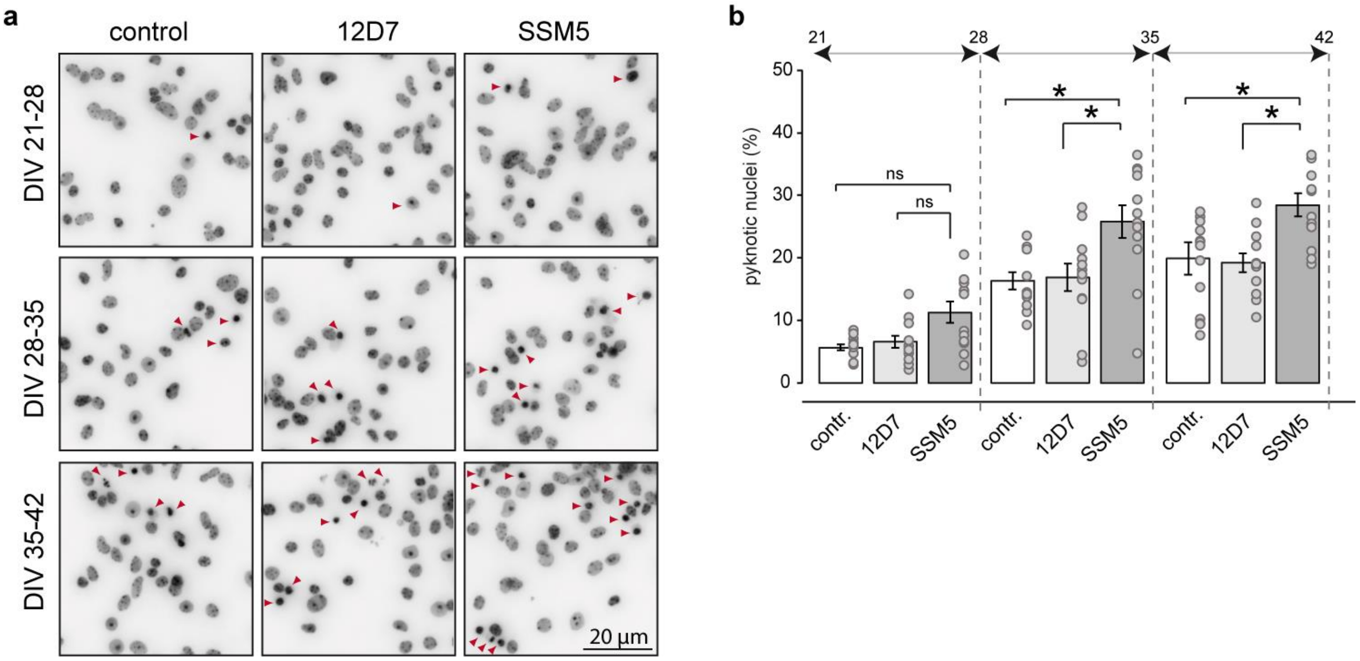

Figure S3. SSM5 antibody increases cell death in late cultures. (**a**) Inverted microscopy images of non-treated control cultures (left panels), vs. cultures treated for one week with 12D7 (middle panels) or SSM5 (right panels) by using staining performed with Hoechst 33258. Red arrowheads indicate abnormal nuclei exhibiting chromatin condensation (pyknosis) or fragmentation (karyorrhexis). Scale bar: 20 µm. (**b**) Data show the percentage of abnormal nuclei (pyknosis or karyorrhexis) of total number of cells. Four independent experiments (technical replicates = 3), Mean ± SEM. One-way ANOVA with Tukey multiple comparison showed significant differences between SSM5 vs 12D7 vs control groups only when treatment was performed in late cultures (DIV 28-42); ns, non-significant; * $p < 0.05$.
